# A theoretical and generalized approach for the assessment of the sample-specific limit of detection for clinical metagenomics

**DOI:** 10.1016/j.csbj.2020.12.040

**Published:** 2020-12-26

**Authors:** Arnt Ebinger, Susanne Fischer, Dirk Höper

**Affiliations:** a Institute for Diagnostic Virology, Friedrich-Loeffler-Institut, Federal Research Institute for Animal Health, Südufer 10, 17493 Greifswald–Insel Riems, Mecklenburg-Western Pomerania, Germany; b Institute of Infectology, Friedrich-Loeffler-Institut, Federal Research Institute for Animal Health, Südufer 10, 17493 Greifswald–Insel Riems, Mecklenburg-Western Pomerania, Germany

**Keywords:** Metagenomics, Next-generation sequencing, Detection limit, Sensitivity, Bernoulli process, qPCR

## Abstract

Metagenomics is a powerful tool to identify novel or unexpected pathogens, since it is generic and relatively unbiased. The limit of detection (LOD) is a critical parameter for the routine application of methods in the clinical diagnostic context. Although attempts for the determination of LODs for metagenomics next-generation sequencing (mNGS) have been made previously, these were only applicable for specific target species in defined samples matrices. Therefore, we developed and validated a generalized probability-based model to assess the sample-specific LOD of mNGS experiments (LOD_mNGS_). Initial rarefaction analyses with datasets of Borna disease virus 1 human encephalitis cases revealed a stochastic behavior of virus read detection. Based on this, we transformed the Bernoulli formula to predict the minimal necessary dataset size to detect one virus read with a probability of 99%. We validated the formula with 30 datasets from diseased individuals, resulting in an accuracy of 99.1% and an average of 4.5 ± 0.4 viral reads found in the calculated minimal dataset size. We demonstrated by modeling the virus genome size, virus-, and total RNA-concentration that the main determinant of mNGS sensitivity is the virus-sample background ratio. The predicted LOD_mNGS_ for the respective pathogenic virus in the datasets were congruent with the virus-concentration determined by RT-qPCR. Theoretical assumptions were further confirmed by correlation analysis of mNGS and RT-qPCR data from the samples of the analyzed datasets. This approach should guide standardization of mNGS application, due to the generalized concept of LOD_mNGS_.

## 1 Introduction

Metagenomic next-generation sequencing (mNGS) is a powerful tool to identify the DNA or RNA of novel or unexpected pathogens in a single-assay. It enables a relatively unbiased detection of all organisms present in a sample, including viruses, bacteria, fungi, and parasites [1]. It has therefore a great potential to fill the gap of detecting undiagnosed causative agents in diseased patients [2], [3], [4]. Routine molecular diagnostic methods like real-time quantitative PCR (qPCR) are highly sensitive, specific and can be standardized [5]. However, the specificity hampers the detection of newly emerging pathogens or distant relatives, like the severe acute respiratory syndrome coronavirus 2 (SARS-CoV-2) and the variegated squirrel bornavirus 1 (VSBV-1) [6], [7]. Additionally, by qPCR only those pathogens can be detected that are specifically targeted. Unexpected pathogens are missed [8]. This gap of diagnosis can lead to a fatal outcome for patients, due to a delayed development and/or implementation of clinical intervention strategies, like vaccination, medication, treatment, and quarantine. In this respect, mNGS is increasingly applied in clinical settings [9]. Technological and bioinformatics advances made it even more attractive [10], [11], [12], [13], [14]. In recent years, ring-trials of bioinformatics pipelines [15], [16] and clinical retro- and prospective studies were performed focusing on proof-of-concept, turnaround-times, accuracy, thresholds to prevent false-positive calls, quality metrics, and analytical and diagnostic specificity and sensitivity [17], [18], [19], [20].

Sensitivity is one of the major factors to assess the power of a diagnostic method. At the first glance, the sensitivity of mNGS is determined by the amount of sequenced reads. Thus, the more reads are sequenced, the further the sensitivity increases. However, the selection of the required data depth has been based mainly on economic factors and empirical and ad-hoc heuristic models, resulting in published datasets that range from 5 to 24 mio reads [17], [18], [19], [21], [22]. Especially for tissue samples, the unbiased sequencing usually results in high background levels of often >99%, which is an inherent disadvantage of mNGS, limiting the analytical sensitivity at constant data depths [22]. To address this issue, targeted pathogen enrichment techniques and host-depletion have been applied [23], [24], [25]. However, they are expensive, complex, and not available for every host or pathogen and moreover do not support the detection of hitherto unknown pathogens. The heterogenic composition of host and pathogen is consequently a key problem in mNGS analysis. Low levels of pathogen reads further complicate the differentiation from commensals and contaminants. Hence, data interpretation has been supported by statistical assessment (z-scores) [26] or methodical parameters, for example calculation of the pathogen reads per million (rpm) [18], to make positive calls based on the pathogen read numbers and proportions. Furthermore, the detection rate is influenced by the genome size of the specific target. In coverage theories, the genome size determines the necessary sequencing effort. To achieve equal sequence depth, a higher sequence data input into assembly is needed for larger genomes than for smaller genomes. Likewise, the detection of a single sequencing read is more likely to come from a large genome rather than a small one at uniform abundance levels [27], [28]. The detection of a species out of the specimen is thus dependent on its abundance, the relative genome size, and the data depth [27]. Therefore, mNGS design should be aware of these factors to find the needle in the metagenome haystack [29], since low abundant pathogens have also been linked to severe diseases [30], [31].

So far, sensitivity assessments of mNGS have been made by comparison with routine methods at qualitative or semi-quantitative levels (Cq values) and by spiking a collection of pathogens in serial dilutions in a specific sample matrix [18], [20], [22], [32], [33]. However, due to the core property of mNGS to detect all nucleic acids with nearly identical probability, a generalization of these pathogen/matrix combination specific results is not possible. Thus, the definition of a limit of detection (LOD) for mNGS (LOD_mNGS_), as applied for other routine methods, is hampered due to the many variables influencing the sensitivity.

Hence, the aim of this study was the development and validation of a pathogen/matrix independent generally applicable mathematical model to assess the detection limit of mNGS experiments. This approach should guide standardization of mNGS application. Therefore, we developed and validated a straightforward analytical tool to assess the sample-specific LOD_mNGS_, which is critical for the routine application of mNGS in the clinical diagnostic context.

## 2 Experimental procedures

### 2.1 Samples and datasets

The study included 30 disease-associated samples and the respective datasets from human and animal cases (Table 1), confirmed by RT-qPCR and mNGS from total RNA. Briefly, five samples originated from brain material of human fatal encephalitis cases caused by Borna disease virus 1 (BoDV-1) [8], [31]. Twenty-five samples with different sample matrices, including lung, brain, heart, liver, and spleen were derived from various host species infected with rustrela virus (RusV) [34], a pegivirus (PGV), or with West Nile virus (WNV) lineage 2 [35], respectively. In the analysed mNGS datasets, virus-specific reads were identified by assembler/mapping analysis after quality and adapter trimming implemented in the 454 software suite (v3.0; Roche). The quality of the library and dataset was checked using FastQC [36] and R-packages bioanalyzeR [37] and qrqc [38] in R-Studio [39] with R (v4.0.2; [40]). Subsequently, the percentage of the respective target virus in the dataset was calculated from the number of virus-specific reads and the total number of reads of that dataset.

**Table 1:** Sample and sequencing information.

Viral target	Library ID	Host	Tissue	Total RNA	RT-qPCR	mNGS		
				(ng/µl)	(Cq value)	total reads	target virus reads	target virus percentage
BoDV-1	lib02012	Human	brain	25.7	15.7	2.69E + 06	2.15E + 04	8.00E – 01
	lib02246	Human	brain	37.6	23.0	7.65E + 06	3.20E + 01	4.18E – 04
	lib02462	Human	brain	17.0	17.8	4.60E + 06	4.61E + 03	1.00E – 01
	lib02557	Human	brain	4.1	19.3	1.15E + 07	1.96E + 04	1.71E – 01
	lib02558	Human	brain	18.4	20.5	3.93E + 06	2.70E + 01	6.86E – 04
PGV	lib03148	European hamster	lung	136.4	26.8	1.45E + 06	2.10E + 01	1.44E – 03
	lib03150	European hamster	lung	260.2	28.0	1.76E + 06	1.00E + 01	5.67E – 04
RusV	lib03123	Donkey	brain	249.0	26.2	2.65E + 06	1.30E + 01	4.91E – 04
WNV	lib02898	Great Grey Owl	organ pool	598.3	11.3	6.82E + 06	3.97E + 05	5.83E + 00
	lib02914	Goshawk	brain	197.3	15.7	6.67E + 06	3.01E + 04	4.52E – 01
	lib02959	Goshawk	brain	80.3	17.6	7.99E + 06	2.79E + 04	3.49E – 01
	lib03378	Snowy Owl	heart	217.4	21.4	2.71E + 06	8.82E + 02	3.26E – 02
	lib03379	Great Grey Owl	liver	898.5	14.3	3.00E + 06	3.61E + 04	1.20E + 00
	lib03380	Snowy Owl	liver	832.1	12.3	3.87E + 06	2.12E + 05	5.46E + 00
	lib03381	Blue Tit	brain	40.6	21.7	4.38E + 06	2.88E + 04	6.57E – 01
	lib03382	Snowy Owl	liver	715.2	16.6	3.55E + 06	1.84E + 04	5.17E – 01
	lib03415	Snowy Owl	heart	189.9	16.8	2.47E + 06	9.73E + 03	3.94E – 01
	lib03416	Andean Flamingo	heart	119.5	17.2	8.37E + 05	3.36E + 03	4.02E – 01
	lib03417	Goshawk	heart	188.9	17.6	1.05E + 06	3.58E + 03	3.41E – 01
	lib03418	Goshawk	brain	134.7	12.5	2.72E + 06	2.94E + 05	1.08E + 01
	lib03419	Goshawk	brain	535.9	13.0	9.25E + 05	4.04E + 04	4.37E + 00
	lib03420	Goshawk	brain	411.5	16.1	7.98E + 05	5.53E + 03	6.93E – 01
	lib03422	Great Tit	liver/heart	1180.9	11.7	1.23E + 06	1.56E + 05	1.27E + 01
	lib03423	Eurasian Golden Plover	liver/spleen	472.7	19.2	1.58E + 06	1.32E + 04	8.38E – 01
	lib03424	Goshawk	brain	446.0	16.7	1.02E + 06	4.92E + 03	4.81E – 01
	lib03425	Snowy Owl	liver	619.9	14.2	1.57E + 06	3.21E + 04	2.05E + 00
	lib03426	Snowy Owl	liver	513.1	17.2	3.77E + 06	1.17E + 04	3.10E – 01
	lib03449	Humboldt-Penguin	heart	270.7	12.1	2.72E + 06	3.06E + 05	1.12E + 01
	lib03450	Goshawk	brain	291.5	14.5	2.32E + 06	3.15E + 04	1.36E + 00
	lib03451	Horse	spinal cord	34.7	28.2	2.94E + 06	1.80E + 01	6.11E – 04

Abbreviations: BoDV-1, Borna disease virus 1; PGV, Pegivirus, RusV, Rustrela virus; WNV, West Nile virus lineage 2; RNA (total), ribonucleic acid concentration of the sample; RT-qPCR, reverse transcriptase real-time PCR; Cq, quantification cycle; mNGS, metagenomics next generation sequencing.

### 2.2 Wet-lab procedures

Total RNA concentrations were quantified using a Nanodrop ND1000 instrument (Peqlab, Erlangen, Germany). The DNA library concentration was measured by using the Bioanalyzer 2100 (Agilent Technologies, CA, USA). Absolute quantification of the viral RNA and the double-stranded virus cDNA (library) was performed by specific 5′ nuclease RT-qPCR and qPCR, respectively (SensiFAST™ Probe No-ROX One-Step Kit, meridian Bioscience, Tennessee, USA). For BoDV-1, Mix1 targeting the P gene was used [8]. For PGV, we used an in-silico and in-vitro confirmed specific assay. For WNV, the INEID-assay targeting the 5′ untranslated region was used [41]. For RusV, an assay targeting the non-structural gene was used [34]. For absolute quantification, a plasmid or synthetic dsDNA (gBlocks®, Integrated DNA Technologies, Leuven, Belgium) calibration standard was applied in duplicates in ten-fold dilutions series from 1.0E + 06 to 1.0E + 01 copies per µl (c/µl) in concordance with the MIQE Guidelines [42]. RT-qPCR calibration curves for BoDV-1, PGV, WNV, and RusV showed an efficiency between 96.6% and 103.1% with R^2^ ranging from 0.9998 to 1.0 and slope in the range from –3.407 to –3.348. For BoDV-1 RNA only, retrospective absolute quantification was carried out with an external calibration curve. An internal standard was used for normalization between the runs. The qPCR calibration curves for the quantification of target virus fragments in the library showed an efficiency between 100.4% and 103.2% with R^2^ ranging from 0.993 to 1.0 and slope in the range from –3.312 to –3.247. For this, 14 libraries, comprising nine WNV (lib03416 – lib03425), two BoDV-1 (lib02246, lib02462), two PGV (lib03148, lib03150), and one RusV (lib03123) were analyzed.

### 2.3 Rarefaction analysis

Rarefaction analyses were performed initially with the five BoDV-1 metagenomics datasets only (lib02012 to lib02558; Table 1). Reads were mapped along the BoDV-1 reference sequence (NC_001607.1) using the 454 software suite (v3.0; Roche, Mannheim, Germany) to identify reads of viral origin. Complete lists of read accessions of the individual libraries were extracted. Then, random subsets of read accessions of each library comprising 1.0E + 02, 1.0E + 03, 1.0E + 04, 5.0E + 04, 1.0E + 05, 5.0E + 05, 1.0E + 06, 2.0E + 06, and 3.0E + 06 reads were retrieved from these lists using the linux command ‘shuf’. In these subsets, read accessions representing viral reads were identified using the linux command ‘fgrep –f’ and the list of accessions representing reads of known viral origin. For each subset size, analyses were repeated 100 times and for each repetition presence, absence of BoDV-1 as well as the number of BoDV-1 reads were recorded. In case the detection rate (presence or absence of BoDV-1 reads) in a given subset size exceeded 95%, only five repetitions were performed because of the low variation in results.

### 2.4 Reference sequences

For calculations exploring factors that influence the limit of detection, we included the following sequences of RNA virus family representatives: West Nile virus (WNV), NC_001563.2, Flaviviridae; Borna disease virus 1 (BoDV-1), NC_001607, Bornaviridae; Rift Valley fever virus (RVF), NC_014397, NC_014396, NC_014395, Bunyaviridae; Sindbis virus (SINV), NC_001547, Togaviridae; Severe acute respiratory syndrome coronavirus 2 (SARS-CoV-2), NC_045512.2, Coronaviridae; Human coxsackievirus A (CV-A2), NC_038306, Picornaviridae; Measles virus (MV), NC_001498, Paramaxoviridae; Rabies lyssavirus (RABV), NC_001542, Rhabdoviridae; Rubella virus (RuV), NC_001545.2, Matonaviridae; Influenza A virus (IAV), GCA_001343785, Orthomyxoviridae; Hepatitis delta virus (HDV), NC_001653.2, Deltavirus incertae sedis.

## 3 Results

### 3.1 Virus read proportion and dataset size determine virus detection

**Fig. 1:**
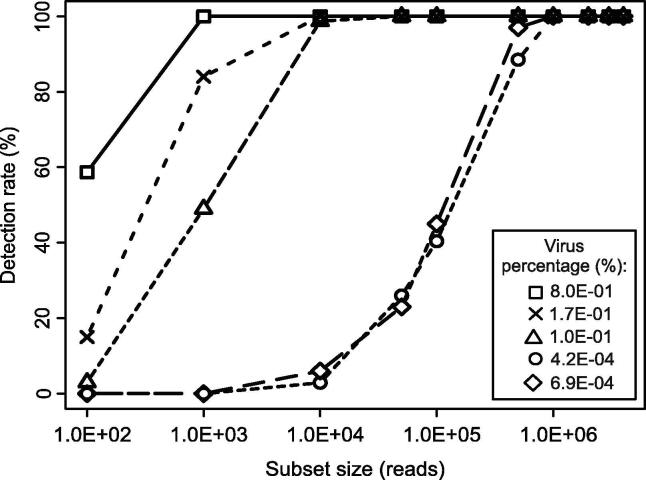
Rarefaction analysis and detection rate of BoDV-1 datasets. Detection rates (%) were calculated by the amount of qualitative BoDV-1 read positive subsamples.

As a starting point for the analyses, we performed a rarefaction analysis. We repetitively determined the detection (presence/absence) of virus reads in data subsets of different size (100 repeats per subset size). From the results of these repetitive drawings, we calculated the positivity rate, i.e. the detection rate of the virus in a given dataset size. For this, we used a set of five datasets generated from BoDV-1-positive samples covering a range of virus read percentages from 6.9E – 04 – 8.0E – 01%. The detection rate of BoDV-1 reads in subsets of these datasets differed (Fig. 1). In subsets of datasets with a low virus read percentage (lib02246 = 4.2E – 04%, lib02558 = 6.9E – 04%) BoDV-1 read detection was possible at a partial dataset size of 1.0E + 06 reads with 100% and at 5.0E + 05 reads with 97% detection rate. At higher virus read percentages in the range of 1.0E – 01 – 8.0E – 01%, BoDV-1 read detection was possible at low partial dataset sizes of 1.0E + 03 reads for lib02012 and 1.0E + 04 reads for lib02462 and lib02557 (detection rates of 100%). The BoDV-1 read amount per partial dataset size increased linearly for all virus read percentages (R^2^ ≥ 0.9851, p ≤ 0.001), despite detection rates of <100% (Extended Data Fig. 1).

### 3.2 Virus detection by mNGS is a Bernoulli process

Based on the results of the rarefaction analyses (=stochastic behavior of virus read detection influenced by dataset size and virus read proportion), we sought a mathematical formula to predict the minimum required dataset size to detect one virus read with a reasonable detection rate. The Bernoulli process describes a discrete stochastic process with only two possible results (presence/absence), coupled with a statement about the probability of occurrence. The equation for the standard Bernoulli process is shown in Equation 1. The notations for the mathematical derivation can be found in Table 2.

*Equation 1: Bernoulli process*

$$P\left(k\right)=\left(\begin{array}{c} n\\ k \end{array}\right)p^{k}{\left(1-p\right)}^{n-k}$$

**Table 2:** Mathematical notations of the Bernoulli formula.

Variable	Meaning
n	Number of trials (size of dataset)
p	Possibility of occurrence (of a viral read; virus read proportion)
k	Number of matches to obtain

The LOD is defined as the lowest quantity that can be detected with reasonable certainty for a given analytical procedure [43]. The chance to detect at least one viral read should be close to 100%. To estimate the dataset size necessary to find one viral read (k = 1) with an event probability of α = 0.99 (0 < α < 1) and a given probability of p, it is necessary to transform Equation 1. Therefore, the arising question was to transform the Bernoulli process to gather an insight into the necessary size of n, i.e. the number of reads sequenced for a library (mNGS). This was done by taking the counter event possibilities into the equation. Following, the natural logarithms were processed and the equation was solved according to equation 2 (Eq. 2). To directly use the virus read proportion of a sample, we set p = $\tilde{p}$/100, where $\tilde{p}$ = virus read percentage.

*Equation 2: Transformed Bernoulli process*

$$n\geq \frac{\ln(1-\alpha )}{\ln(1-\frac{\tilde{p}}{100})}$$

Validation of the transformed Bernoulli formula was performed with $\tilde{p}$ which originated from the mNGS analysis of the 30 trimmed and quality checked datasets from diseased animals and humans (Table 1, Extended Data Figs. 2, 3, and 4). The $\tilde{p}$ from the mNGS and assembler/mapping analysis resulted from the number of virus-specific reads and the total number of all sequenced reads of a library. One-hundred subsamples from the total read accession numbers of the libraries were taken respectively with replacement and were compared to the accession list of mapped virus reads. The mean accuracy of Eq. 2 to predict dataset size n for a virus read was 99.1% within the range of 93.0 to 100.0% at a qualitative level (Table 3). We proved the assumption of k = 1 virus read of Eq. 2 by counting the amount of the respective virus reads in the subsets. This resulted in k = 4.5 ± 0.4 reads in n (Table 3). As a cross check of Eq. 2, we reconstructed the number of virus reads from the mNGS analyses. To do this, we divided the individual dataset sizes (Table 1) by the calculated n (Table 3). We included k = 4.5 as a multiplication factor in Equation 3, since k ≠ 1.

**Table 3:** Validation of the transformed Bernoulli formula.

*Viral target*	*ID*	*n*	*Accuracy (%)*	*Viral reads*	
				*Mean*	*SD*
*BoDV-1*	lib02012	559	99	4.7	2.2
	lib02246	1,101,151	100	4.7	2.0
	lib02462	4603	93	2.8	1.8
	lib02557	2693	100	4.3	2.2
	lib02558	670,830	100	4.4	1.9
*PGV*	lib03148	318,891	100	4.5	1.9
	lib03150	807,922	100	4.4	1.7
*RusV*	lib03123	939,828	100	4.5	1.7
*WNV*	lib02898	77	98	4.7	2.1
	lib02914	1017	99	4.5	2.2
	lib02959	1317	100	4.5	2.4
	lib03378	14,144	97	4.5	2.1
	lib03379	381	100	4.5	1.7
	lib03380	82	100	4.8	2.0
	lib03381	699	99	4.8	2.4
	lib03382	888	100	5.0	2.3
	lib03415	1166	99	4.7	1.9
	lib03416	1144	97	4.5	2.0
	lib03417	1349	98	4.7	2.1
	lib03418	40	100	4.3	1.8
	lib03419	103	100	4.7	2.2
	lib03420	662	100	4.7	2.1
	lib03422	34	100	4.3	1.8
	lib03423	547	99	4.8	2.1
	lib03424	955	100	4.2	1.9
	lib03425	223	98	4.7	2.1
	lib03426	1481	99	5.0	2.0
	lib03449	39	100	4.3	2.0
	lib03450	337	99	4.6	2.2
	lib03451	754,944	100	4.8	1.5
	Mean		**99.1**	**4.5**	**2.0**
	SD		***1.5***	**0.4**	**0.2**

Abbreviations: BoDV-1, Borna disease virus 1; PGV, Pegivirus, RusV, Rustrela virus; WNV, West Nile virus lineage 2; n, theoretically required dataset size for 1 virus read; SD, standard deviation.

*Equation 3: Recovery of virus read numbers*

$$\tilde{p}\;\left(r\right)=\left(\frac{r}{n}\right)*k,$$

where r = actually available dataset size, n = theoretically required dataset size for ≥1 virus read (Eq. 2), and k = multiplication factor. The recovery rate was 97.99% (median; Extended Data Fig. 5).

### 3.3 Modelling factors that impact mNGS sensitivity

As mentioned above, empirical data shows that the detection of a species depends on its abundance, the relative genome size, and the dataset size. We used R Studio [39] to investigate the influence of these factors on mNGS sensitivity. To be able to apply Eq. 2 for the prediction of the necessary sequencing effort, we approximate $\tilde{p}$ as the ratio of the amounts (in g) of viral RNA and total RNA in the sample. We approximated $\tilde{p}$ from the amount of viral RNA calculated from the virus genome copy number and the amount of total RNA as determined photometrically with Equation 4 (Eq. 4).

*Equation 4: Prediction of* $\tilde{p}$

$${\overset{∼}{p}}_{i}=\frac{100*nt*340\;Da*\;1.6605402E\;–\;15\;ng/Da*c}{\mathrm{total}\;RNA\;concentration}$$

where i = virus genome copies, nt = virus genome size, 340 Da = mean weight of one RNA nucleotide in Dalton, 1.6605402E – 15 = weight of one Da in nanogram, and c = i/µl.

**Fig. 2:**
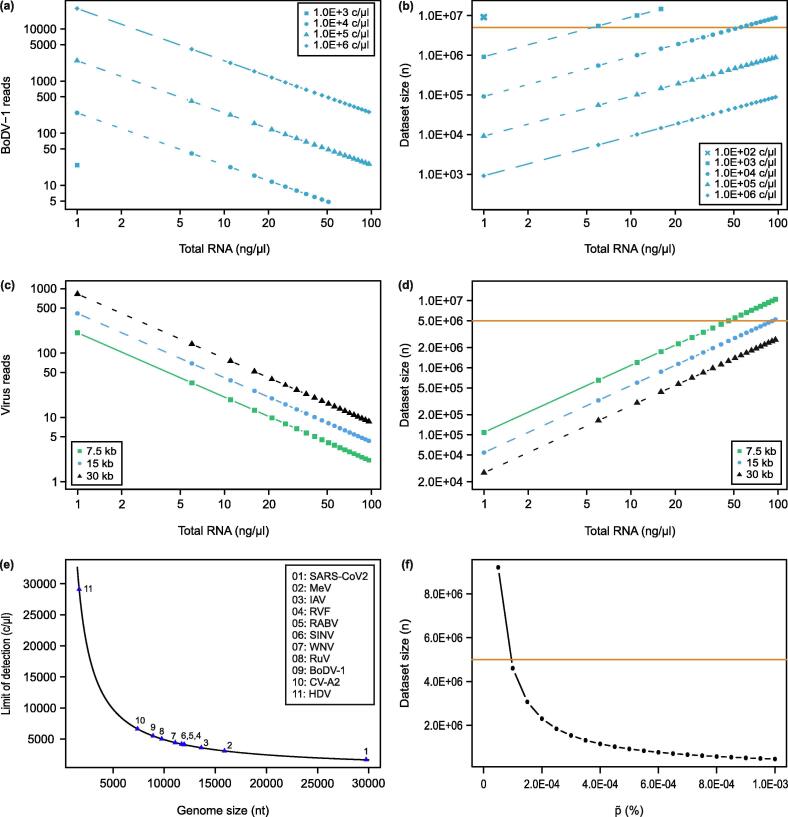
Theoretical evaluation of impact factors for mNGS analytical sensitivity. (a) Amount of BoDV-1 reads in relation to the total RNA- and virus-concentration at dataset size 5.0E + 06 reads. No BoDV-1 reads were calculated for 1.0E + 02 c/µl. (b) Minimal dataset size (n) required to detect at least one BoDV-1 read depending on the RNA- and virus-concentration. Threshold (orange line) was set at 5.0E + 06 read dataset size. (c) Amount of virus reads depending on the genome length and RNA-concentration. Virus concentration at 1.0E + 04 c/µl and data set size at 5.0E + 06 reads. (d) Minimal dataset size (n) required to detect at least one virus read depending on the genome length at a virus-concentration of 1.0E + 04 c/µl. Threshold (orange line) was set at 5.0E + 06 read dataset size. (e) Limit of detection of mNGS depending on the genome length of RNA viruses. Dataset size at 5.0E + 06 reads and RNA-concentration at 30 ng/µl. Severe acute respiratory syndrome coronavirus 2, SARS-CoV-2; measles virus, MeV; Influenza A virus, IAV; Rift Valley fever virus, RVFV; Rabies lyssavirus, RABV; Sindbis virus, SINV; West Nile virus, WNV; Rubella virus, RuV; Borna disease virus 1, BoDV-1; Coxsackievirus A, CV-A2; Hepatitis delta virus, HDV. (f) Minimal dataset size (n) required to detect at least one pathogen read depending on $\tilde{p}$. Threshold (orange line) was set at 5.0E + 06 read data depth. (For interpretation of the references to colour in this figure legend, the reader is referred to the web version of this article.)

Applying Eq. 2 in combination with Eq. 3 and Eq. 4, we modeled $\tilde{p}$ in dependence of different factors but with constant α = 0.99 (Fig. 2). First, we investigated the effect of $\tilde{p}$ on the expected number of BoDV-1 reads in a dataset of defined size (r = 5.0E + 06 reads) in dependence of the virus copy number per µl and the total RNA concentration. To assess the sensitivity, a tenfold serial dilution of 1.0E + 00 to 1.0E + 06 c/µl of the BoDV-1 genome (8910 nt, NC_001607) was used, while RNA concentration was increased (1 to 100 ng/µl) (Fig. 2a). As Fig. 2a shows, the expected number of BoDV-1 reads differed within and between virus concentrations, showing a decrease in virus reads with a simultaneous increase in total RNA concentration. To illuminate qualitative diagnostic aspects, we calculated the necessary dataset size n for the same dependencies as in Fig. 2a with an upper cut-off for dataset size set at 1.5E + 07 reads (Fig. 2b). This showed that with copy numbers higher than 1.0E + 05 c/µl, BoDV-1 was detectable independently of the background, i.e. at every $\tilde{p}$. On the contrary, with BoDV-1 copy numbers below 1.0E + 04 c/µl, virus reads were only detectable at total RNA concentrations lower than approx. 50 ng/µl (Fig. 2b). With a BoDV-1 copy number below 1.0E + 02 c/µl, no detection was possible with a dataset size of 5.0E + 06 reads (Fig. 2b).

In order to generalize the model, we investigated the influence of the genome size on the virus read numbers at a given dataset size (Fig. 2c) and the necessary dataset size (Fig. 2d). For these analyses, we repeated the calculations with representative genome sizes for small, medium and large RNA virus genomes (7.5 kb, 15 kb, and 30 kb) at a concentration of 1.0E + 04 c/µl. As Fig. 2c shows, the number of virus reads that can be expected in a dataset of 5.0E + 06 reads depends on the genome size. The detection of a read from a virus with a small genome (7.5 kb) size required higher dataset sizes (n) than for larger viruses (15 and 30 kb; Fig. 2d).

To assess the meaningfulness of the result obtained with a certain assay, the limit of detection (LOD) of that assay needs to be defined. Although in practice the LOD of qPCR depends on the specific assay, theoretically the LOD of qPCR is at the genome copy number of 3 c/µl but independent of the genome size. As shown above, the sensitivity of mNGS depends on both virus copy number and virus genome size. In order to investigate the limit of detection for mNGS analysis, we calculated the minimum virus genome copy number that allows for the detection of a virus in a dataset of 5.0E + 06 reads generated from a sample with 30 ng/µl total RNA. Specifically, we further examined the effect of the genome size (1.5 kb to 30 kb) on the detection limit of an mNGS analysis. For this, we calculated the LOD of an mNGS analysis as follows: For each $\tilde{p}$_i_ (1.0E + 00 ≤ i ≤ 1.0E + 06 c/µl; Eq. 4), the theoretically necessary minimal dataset size n was calculated according to Eq. 2. The LOD is then defined as the minimal c/µl for which 1 viral read can be expected in a dataset of 5.0E + 06 reads. As shown in Fig. 2e, the LOD varies among the genome sizes. The LOD for the very large SARS-CoV-2 (1686 c/µl) and the very small HDV (29106 c/µl) differs 17.3 times from each other.

To evaluate the sensitivity independent of the pathogen (genome size and copy number) and the total nucleic acid concentration, we calculated n (the necessary dataset size to detect 1 viral read) for a range of $\tilde{p}$ (5.0E – 05 – 1.0E – 03%). In this analysis, we observed an exponential decrease in the required dataset size n (Fig. 2f). For all $\tilde{p}$≥ 0.0001% the pathogen was detectable with a dataset size of 5.0E + 06 reads. For $\tilde{p}$< 0.0001% a higher amount of sequenced reads were necessary, indicating that in theory the sensitivity can be scaled by scaling the dataset size.

### 3.4 $\tilde{p}$_RT-qPCR_ and $\tilde{p}$_mNGS_ are significantly correlated

**Fig. 3:**
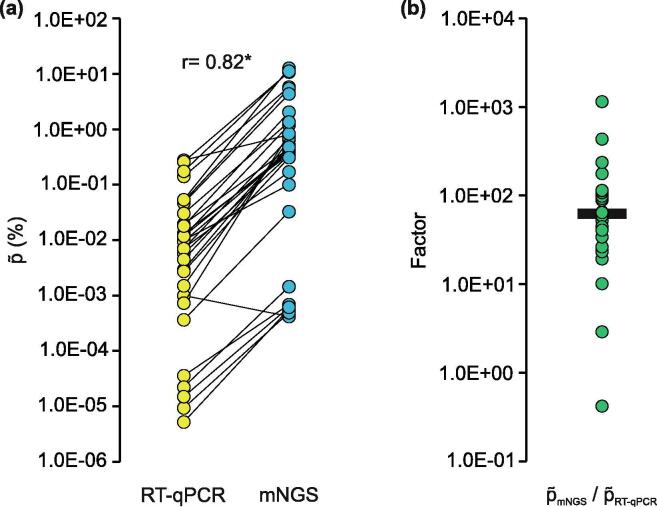
Comparison of RT-qPCR and mNGS derived virus to background ratios. (a) $\tilde{p}$ were determined by mNGS analysis (proportion of virus reads to total reads) and absolute quantification by RT-qPCR (proportion of virus RNA in ng/µl to the total RNA concentration); r = 0.82, **p < 0.0001. (b) Discrepancy of mNGS and RT-qPCR derived $\tilde{p}$ displayed as the factor of $\tilde{p}$_mNGS_ / $\tilde{p}$_RT-qPCR_; median = 61.7.

As a proof-of-concept that for mNGS analysis $\tilde{p}$ is defined as the ratio of the mass of viral nucleic acids and total RNA, we compared $\tilde{p}$_mNGS_ and $\tilde{p}$_RT-qPCR_. To this end, we calculated $\tilde{p}$ from the quantitative RT-qPCR results by Eq. 4. For these calculations the genome sizes of the individual viruses (BoDV-1, 8910 nt; RusV, 9322 nt; WNV, 11080; PGV, 11,520 nt) were used. The calculated $\tilde{p}$_RT-qPCR_ correlated highly significant with the $\tilde{p}$_mNGS_ (r = 0.82, p < 0.0001). Unexpectedly, with a single exception (BoDV-1 in lib02246; Fig. 3a, Extended Data Table 1) $\tilde{p}$_mNGS_ were higher (median 61.7 times, IQR 35.8 – 107.8) than $\tilde{p}$_RT-qPCR_ (Fig. 3b). Therefore, to trace the source of this deviation, we determined $\tilde{p}$_Library_ in the sequencing-ready libraries. To this end, we analyzed 14 libraries by qPCR and Agilent Bioanalyzer. For the calculations of $\tilde{p}$_Library_, we modified the conversion factor for 340 Da for RNA into 660 Da for dsDNA in Eq. 4 and put the amount of qPCR target molecules in relation to the DNA library concentration. For a subset of five libraries, we observed an increase of $\tilde{p}$ in the library ($\tilde{p}$_Library_; median = 1.2E – 03%) in comparison with $\tilde{p}$_RT-qPCR_ (median = 2.2E – 05%; Extended Data Fig. 6). This coincided with $\tilde{p}$_mNGS_ of these libraries. However, the same libraries had an increased $\tilde{p}$ in comparison to $\tilde{p}$_mNGS_ (median = 5.7E – 04%). Unfortunately, nine WNV libraries had to be excluded from this analysis of $\tilde{p}$_Library_ due to methodical constraints. Here, the RT-qPCR assay is located at the 5′-terminus of the genome, which is not converted efficiently during library preparation, as displayed by qPCR data and genome coverage analyses (data not shown). Hence, no reliable determination of $\tilde{p}$_Library_ was possible.

### 3.5 Detection limits of mNGS appear primarily determined by total RNA concentration

**Fig. 4:**
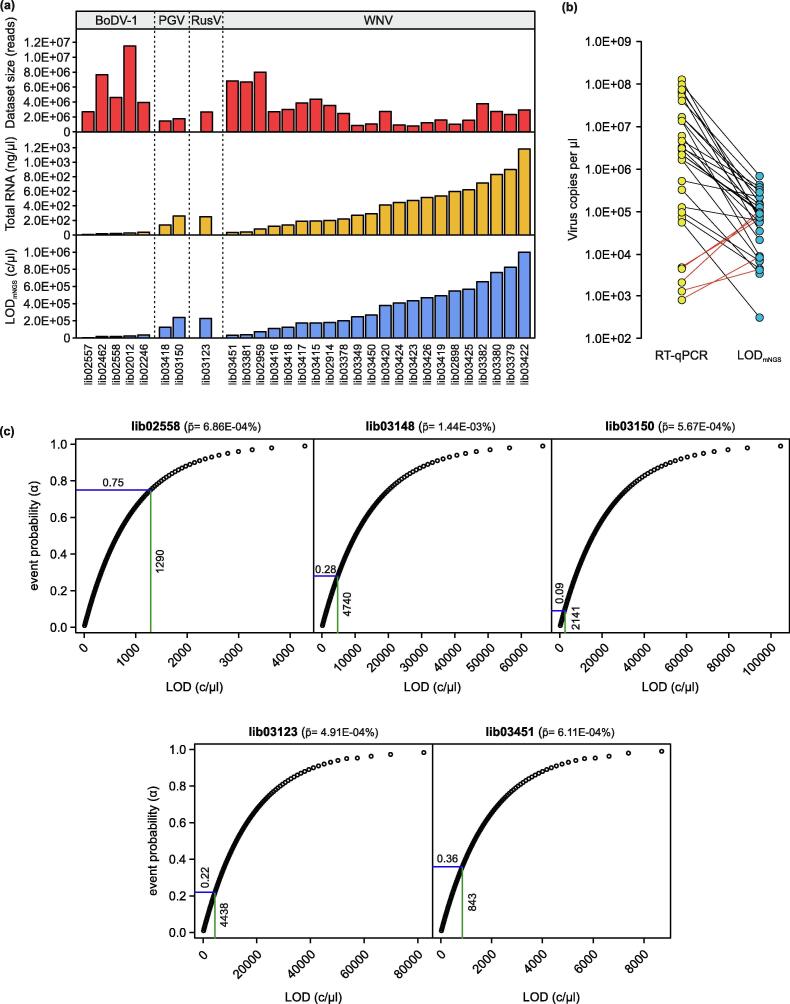
Calculation of the sample-based detection limit from datasets. (a) Key characteristics of the analysed samples and datasets. Upper panel, dataset size; mid panel, total RNA concentration; lower panel, LOD_mNGS_. Calculations were performed with 30 datasets originated from diseased animals (Table 1) by combination of equations 2, 3, and 4. (b) Comparison of the LOD_mNGS_ (c/µl) and viral genome copy numbers (c/µl) as determined by RT-qPCR quantification of the samples. Red lines display the samples where LOD_mNGS_ > RT-qPCR c/µl. (c) Probability-based calculation of LOD_mNGS_ for the five samples labelled red in (b). Horizontal lines represent the adjusted α to meet the criteria LOD_mNGS_ < copy number determined by RT-qPCR. Vertical lines represent the calculated LOD_mNGS_ (c/µl) with the adjusted α. (For interpretation of the references to colour in this figure legend, the reader is referred to the web version of this article.)

As outlined above, in published studies the sensitivity of mNGS is often tried to define by comparison with routine diagnostic methods. Therefore, here we conducted a systematic comparison of the LODs calculated from mNGS data with the virus genome copy numbers determined by RT-qPCR from the identical sample. To this end, we calculated the LOD_mNGS_ using Eq. 2 and its modification (Eq. 4, calculation of LOD) to the datasets used in this study (Table 1). The LOD_mNGS_ calculated for the individual libraries differed, apparently rather in relation to the total RNA concentration than to the amount of sequenced reads or virus species (Fig. 4a; Extended Data Table 1). LOD_mNGS_ values were considered plausible if lower than or equal to the virus copy numbers per µl as determined by RT-qPCR. This was true in 25/30 cases (Fig. 4b, Extended Data Table 1). Although the detection of virus concentrations below the calculated LOD is by definition very unlikely, this was observed for five libraries containing different viruses (lib02558, BoDV-1; lib03148 and lib03150, PGV; lib03123, RusV; lib03451, WNV; Fig. 4b and Extended Data Table 1). For these five samples, we recalculated the LOD_mNGS_ for the different event probabilities for α = 0.01 to 0.99 (stepwise increase of 0.01) and compared it to the c/µl of RT-qPCR (Fig. 4c). In all these cases, the recalculated LOD_mNGS_ was plausible according to the definition above, albeit with reduced α in the range of 0.09 and 0.75 (Fig. 4c).

### 3.6 $\tilde{p}$_mNGS_ and LOD_mNGS_ are significantly correlated with RT-qPCR values

**Fig. 5:**
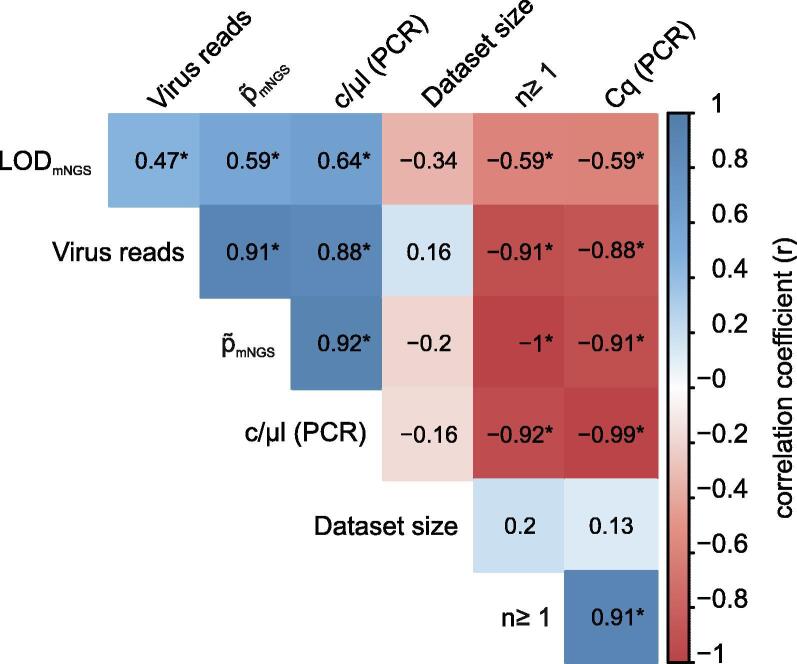
Correlation matrix of RT-qPCR impact factors for mNGS sensitivity. Values derived from samples and formula were compared. The correlation coefficient is displayed from + 1 to −1 (positive and negative correlation). An asterisk indicates p < 0.01.

We conclusively examined the correlation between the various sample and dataset characteristics examined above. To this end, values were log-transformed prior to the calculation of spearman correlations and p-values with the rcorr() function of the Hmisc package [44] in R Studio. The correlation matrix was created with the corrplot package [45]. In this analysis we included Cq-values, virus copy numbers calculated from RT-qPCR values (Cq, c/µl), dataset size, number of virus reads, $\tilde{p}$_mNGS_, n, and LOD_mNGS_. Inverse correlation of semi-quantitative (Cq) and absolute quantitative (c/µl) RT-qPCR values were observed (Fig. 5). As Fig. 5 shows, this analysis revealed highly significant (p < 0.01) correlations of RT-qPCR values and mNGS (viral reads, $\tilde{p}$_mNGS_) and formula-derived values (n, LOD_mNGS_), respectively. Obviously, the correlation between mNGS and formula-derived values is due to the dependency of the formula derived values from the mNGS data. None of the categories had significant correlation with the dataset size. This correlation analyses clearly shows that the calculation of LOD_mNGS_ and the necessary dataset size n is possible and yields meaningful results. These allow the assessment of the mNGS based detection limit depending on $\tilde{p}$.

## 4 Discussion

We developed a straightforward probability-based mathematical approach to test the assignment of the individual detection limit per sample for mNGS analysis. We followed a sample matrix-independent approach to preserve the advantageous non-specificity of mNGS in pathogen detection and at the same time make a statistical statement about the probability of virus detection at a certain data depth. The assessment of an mNGS result must always take into account the specific detection limit of the analysis for a certain pathogen and the analysis of specific parameters (total nucleic acid input, expected pathogen genome size, dataset size; compare Fig. 2e). Our model incorporates the hitherto known factors influencing LOD_mNGS_, whereby valuable information can be derviated for the assessment of mNGS experiments and related expectations. The expression of LOD_mNGS_ in copies per microliter enables comparison with RT-qPCR derived concentrations. To the best of our knowledge, we showed for the first time a direct relationship between the ratio of viral and total RNA and its ratio after mNGS analysis.

Rarefaction analysis of the BoDV-1 datasets showed that the relationship between virus detection rate and dataset depths depends on the virus read percentage $\tilde{p}$. Therefore, we concluded that the presence or absence of a virus read at a certain, minimal dataset size follows a Bernoulli distribution, a discrete probability distribution with binomial results. We transformed the formula of the Bernoulli process into Eq. 2 to calculate the dataset size required for virus detection with a given probability. We set α = 0.99 (99%) to detect the virus read with a probability close to 100%. The introduction of a probability for the detection limit for mNGS is thus in line with the general definition of LOD [43]. The verification of Eq. 2 with datasets from diseased animals and humans showed a high accuracy and repeatability, confirming our probability based approach. However, the accuracy of n (minimal dataset size for the detection of one virus read) was influenced by the accurate determination of the virus-background-ratio, designated $\tilde{p}$. We argue that an incorrect assignment of virus reads in the determination of $\tilde{p}$ for BoDV-1 reads in lib02462 resulted in a slightly reduced accuracy (93%) and 2.8 ± 1.8 virus reads. In Eq. 2, we set k = 1 virus read to calculate n. Indeed, in datasets of size n, a mean of 4.5 ± 0.4 reads were counted. Actually, dataset size n is calculated for k = 1. At last, we confirmed the applicability and correctness of Eq. 2 and k = 4.5 by recovering the actual virus read numbers with an accuracy of 97.99%.

We demonstrated that the critical factor of the mNGS sensitivity is $\tilde{p}$. We observed a logarithmic relationship of $\tilde{p}$ and n, indicating that a pathogen abundance level of $\tilde{p}$ > 0.001% is already reliably detectable within a dataset of 5.0E + 06 reads (Fig. 2f). Due to the logarithmic relation of $\tilde{p}$ and n, lower $\tilde{p}$ require disproportionately large datasets. Interestingly, a log relationship of Cq values and mapped reads of viral pathogens in nasopharyngeal swabs has already been observed [32]. This observation also fits with published [30] findings that the selection of a suitable sample is critical for the success of mNGS analyses.

However, $\tilde{p}$ is a relative value. The nucleic acid amount of larger viruses is naturally higher than that of a small one at the same concentration, i.e. genome copy number (c/µl). The effect of the genome size and the probability of occurrence of a single species read has already been reported [27]. Moreover, the genome size has already been taken into account in the normalization of read counts (RPKM [20], VTMK [46]) and in experimental planning for assembly approaches [28]. We also observed an effect of the genome size on LOD_mNGS_. The LOD_mNGS_ decreased with increasing genome sizes (Fig. 2e). The LOD_mNGS_ for SARS-CoV-2 was 17.3 times lower than for HDV. When comparing large DNA viruses, bacteria or parasites, the impact of genome size on the differences in the LOD will be more pronounced. Additionally, the basic assumption of our calculations and those from RT-qPCR quantification relies on linking a target read or amplicon of small size to a genomic equivalent, neglecting differences in genome coverage as potentially caused by transcriptional gradients or the expression of subgenomic RNA found in several species [47]. Furthermore, we show that the virus-concentration is not a reliable indicator of mNGS sensitivity (compare Fig. 2a, 2b). With decreasing $\tilde{p}$, i.e. increasing background, the same virus genome copy number can lead to different amount of virus reads and required dataset size for detection. In absolute read numbers, in a dataset of 5.0E + 06 reads generated from a sample with 50 ng/µl total RNA and a virus concentration of 1.0E + 04 c/µl one would receive 5 BoDV-1 reads while at 1 ng/µl total RNA with the same virus concentration, the same dataset would comprise approx. 400 viral reads (Fig. 2a, 2b). Consequently, with a virus concentration of 1.0E + 04 c/µl and 1 ng/µl total RNA only 1.0E + 05 reads (minimal dataset size n) are needed for detection of BoDV-1, whereas 5.0E + 06 reads are needed at 50 ng/µl total RNA. The effect of high and low background is well known [17], [18], [19], [33], [48]. Consequently, highly abundant pathogens are more obvious than low-abundant pathogens and the differentiation to a contaminant becomes more important [49], [50]. At a low pathogen read and abundance level, assembly approaches may fail or threshold criteria used to differentiate clinically relevant pathogens from contaminants may not be met [18], [19], but even a single pathogen read should be reviewed carefully and should not be rejected per se [19], [30]. Nevertheless, it is of course not advisable to derive a diagnosis or even a clinical treatment strategy based on single or few reads. Especially single or low abundant pathogen reads need to be reviewed carefully and a false assignment e.g. due to low-complexity regions, has to be excluded by a data analyst. However, knowledge of LOD_mNGS_ can help to assess and rank the obtained results and provide valuable information to base the decision on whether or not it is worth following up the findings.

Deducing $\tilde{p}$ from absolute quantitative RT-qPCR is in principle possible (r = 0.82, p < 0.0001, Fig. 3). We also confirmed the correlation of RT-qPCR values and mNGS results (Fig. 5). The observed factor of 61.7 between $\tilde{p}$_mNGS_ and $\tilde{p}$_RT-qPCR_ is presumably a combined effect of different experimental factors: (i) The use of an external DNA standard (according to the original publication [51]) instead of an RNA standard may render the absolute quantification of our RT-qPCR assays somewhat inexact by disregarding the efficiency of the reverse transcriptase [52]; (ii) it is presupposed that a suitable method for measuring the total RNA concentration is applied in order not to flaw the $\tilde{p}$_RT-qPCR_ or LOD_mNGS_ calculations; this is especially true for samples with low biomass (<10 ng); however, we did not observe substantial differences of the low biomass lib02557 (4.1 ng/µl) to all other analyzed libraries (≥17 ng/µl; Table 1, Extended Data Table 1, Fig. 4,); (iii) presumably most importantly, library preparation impacts the finally resulting $\tilde{p}$_mNGS_; it alters the composition of the total nucleic acids by enzymatic modifications including reverse-transcription, fragment end polishing, and adapter ligation. Of course lastly also size selection impacts the composition by removing small and large fragments from the sample during library preparation [10]. To assess the impact of library preparation and distinguish its effect from potential sequencing bias, we determined $\tilde{p}$_Library_ from a set of analyzed libraries. Although due to technical constraints only a subset of the data could be taken into account, it appears that the main difference between $\tilde{p}$_RT-qPCR_ and $\tilde{p}$_mNGS_ is introduced during library preparation. This does of course not rule out differences of viral read proportions in datasets which can derive from different sequencing platforms and their respective library preparation workflows, affecting $\tilde{p}$_mNGS_ [53], [54]. Rather, it can be expected that each workflow from sample to sequence dataset will have its specific factor between $\tilde{p}$_RT-qPCR_ and $\tilde{p}$_mNGS_. Therefore, further studies are needed to identify such factors to adjust our model and increase its level of precision.

In Fig. 4a, we modelled the LOD for 30 datasets that originated from various sample matrices of diseased animals and humans. We calculated the individual LOD_mNGS_ for every sample based on the target virus, total RNA concentration, and dataset size. While LOD_mNGS_ increased with increasing total RNA-concentrations, the impact of the dataset size was neglectable. This missing influence of the dataset size may be caused by the selected datasets, although these were randomly selected from available datasets. The accuracy of the calculated LOD remains to be assessed by systematic comparison of mNGS negative but RT-qPCR positive samples. Nevertheless, in 25/30 cases the RT-qPCR derived quantitative values were above the mNGS LOD, supporting the dependencies between sample and LOD_mNGS_ elaborated in this paper. In the remaining cases, LOD_mNGS_ was higher than the concentration derived from RT-qPCR. All these had a $\tilde{p}$ of 1.44E-03 – 6.86E-04 and ≤27 virus reads. We argue that the used data depth for these samples was too low to fulfill the 99% probability requirement for the occurrence of at least one viral read in a data subset. Systematic analysis are needed to evaluate the effect of data depth and probability of detection as well as to validate the predicted and actual LOD.

In previous studies, the detection cut-offs of mNGS have been linked to Cq ~32 and ~36 in nasopharyngeal swabs, aspirates, or sputums for different virus panels [20], [32] or have been evaluated by a serial dilution of a set of pathogens, including human immunodeficiency virus and cytomegalovirus with 313 and 14 copies/ml in CSF samples [18]. Although these results highlight the limitation and power of mNGS, the results are hardly transferable to other matrices and viruses. Additionally, differences in sequencing depths complicate a generalization of the detection limit. A general definition of LOD_mNGS_ seems therefore not suitable but appears rather matrix and pathogen-specific [17], [18], [20], [32]. However, our approach supports the standardization of the mNGS detection limit across matrices and pathogens.

## 5 Conclusion

The assessment of the detection limit is of major interest for the application of shotgun mNGS in clinical laboratories. Therefore, we developed and validated a straightforward analytical tool to assess the sample-specific LOD_mNGS_, considering nucleic acid concentration, genome length, and data depth. For this calculation, we define the total nucleic acid concentration as the background for modeling the LOD_mNGS_. The results of these calculations are congruent with RT-qPCR results. This mathematical and sample matrix independent approach may guide to a more transferable and standardizable LOD for future mNGS experiments.

## CRediT authorship contribution statement

**Arnt Ebinger:** Formal analysis, Investigation, Methodology, Software, Validation, Visualization, Data curation, Writing - original draft, Writing - review & editing. **Susanne Fischer:** Conceptualization, Methodology, Validation, Writing - review & editing. **Dirk Höper:** Conceptualization, Funding acquisition, Methodology, Supervision, Writing - original draft, Writing - review & editing.

## Declaration of Competing Interest

The authors declare that they have no known competing financial interests or personal relationships that could have appeared to influence the work reported in this paper.
