# Supplementary material for: A theoretical and generalized approach for the assessment of the sample-specific limit of detection for clinical metagenomics
Source: Comput Struct Biotechnol J. 2020 Dec 26;19:732–42. doi: 10.1016/j.csbj.2020.12.040 (PMC7822954; doi:10.1016/j.csbj.2020.12.040)
Supplement: Supplementary data 1 [file mmc1.docx]

**Supplement:**

**Extended Data Table 1.** Detection limits and virus-to-background (p̃) ratio calculation by mNGS and RT-qPCR.

| *Viral target* | *ID* | p̃*_mNGS_* | p̃*_RT-PCR_* | *LOD_mNGS_ (c/µl)* | *RT-qPCR (c/µl)* |
| --- | --- | --- | --- | --- | --- |
| BoDV-1 | lib02012 | 8.00E-01 | 2.77E-01 | 8.76E+03 | 1.41E+07 |
|  | lib02246 | 4.18E-04 | 9.99E-04 | 4.50E+03 | 7.47E+04 |
|  | lib02462 | 1.00E-01 | 9.90E-03 | 3.38E+03 | 3.35E+05 |
|  | lib02557 | 1.71E-01 | 7.19E-03 | 3.27E+02 | 5.86E+04 |
|  | lib02558 | 6.86E-04 | 3.57E-05 | 4.28E+03 | 1.30E+03 |
| PGV | lib03148 | 1.44E-03 | 2.22E-05 | 6.65E+04 | 4.84E+03 |
|  | lib03150 | 5.67E-04 | 5.20E-06 | 1.05E+05 | 2.16E+03 |
| RusV | lib03123 | 4.91E-04 | 9.37E-06 | 8.23E+04 | 4.44E+03 |
| WNV | lib02898 | 5.83E+00 | 1.41E-01 | 6.46E+04 | 1.35E+08 |
|  | lib02914 | 4.52E-01 | 1.94E-02 | 2.18E+04 | 6.11E+06 |
|  | lib02959 | 3.49E-01 | 1.33E-02 | 7.39E+03 | 1.70E+06 |
|  | lib03378 | 3.26E-02 | 3.65E-04 | 5.91E+04 | 1.27E+05 |
|  | lib03379 | 1.20E+00 | 1.16E-02 | 2.21E+05 | 1.67E+07 |
|  | lib03380 | 5.46E+00 | 5.07E-02 | 1.58E+05 | 6.74E+07 |
|  | lib03381 | 6.57E-01 | 1.51E-03 | 6.83E+03 | 9.81E+04 |
|  | lib03382 | 5.17E-01 | 2.93E-03 | 1.48E+05 | 3.35E+06 |
|  | lib03415 | 3.94E-01 | 9.48E-03 | 5.66E+04 | 2.88E+06 |
|  | lib03416 | 4.02E-01 | 1.18E-02 | 1.05E+05 | 2.25E+06 |
|  | lib03417 | 3.41E-01 | 5.76E-03 | 1.32E+05 | 1.74E+06 |
|  | lib03418 | 1.08E+01 | 2.61E-01 | 3.65E+04 | 5.63E+07 |
|  | lib03419 | 4.37E+00 | 4.71E-02 | 4.27E+05 | 4.04E+07 |
|  | lib03420 | 6.93E-01 | 7.04E-03 | 3.80E+05 | 4.63E+06 |
|  | lib03422 | 1.27E+01 | 5.40E-02 | 7.07E+05 | 1.02E+08 |
|  | lib03423 | 8.38E-01 | 7.29E-04 | 2.20E+05 | 5.51E+05 |
|  | lib03424 | 4.81E-01 | 4.47E-03 | 3.22E+05 | 3.19E+06 |
|  | lib03425 | 2.05E+00 | 1.80E-02 | 2.91E+05 | 1.78E+07 |
|  | lib03426 | 3.10E-01 | 2.72E-03 | 1.00E+05 | 2.23E+06 |
|  | lib03449 | 1.12E+01 | 1.75E-01 | 7.33E+04 | 7.58E+07 |
|  | lib03450 | 1.36E+00 | 3.05E-02 | 9.25E+04 | 1.42E+07 |
|  | lib03451 | 6.11E-04 | 1.50E-05 | 8.70E+03 | 8.32E+02 |

*Abbreviations: BoDV-1, Borna disease virus 1; PGV, Pegivirus, RusV, Rustrela virus; WNV, West Nile virus lineage 2;* p̃*, virus to background ratio in percent; RT-qPCR, reverse transcriptase real-time PCR; mNGS, metagenomics next generation sequencing; LOD, limit of detection.*

**Extended Data Figures:**


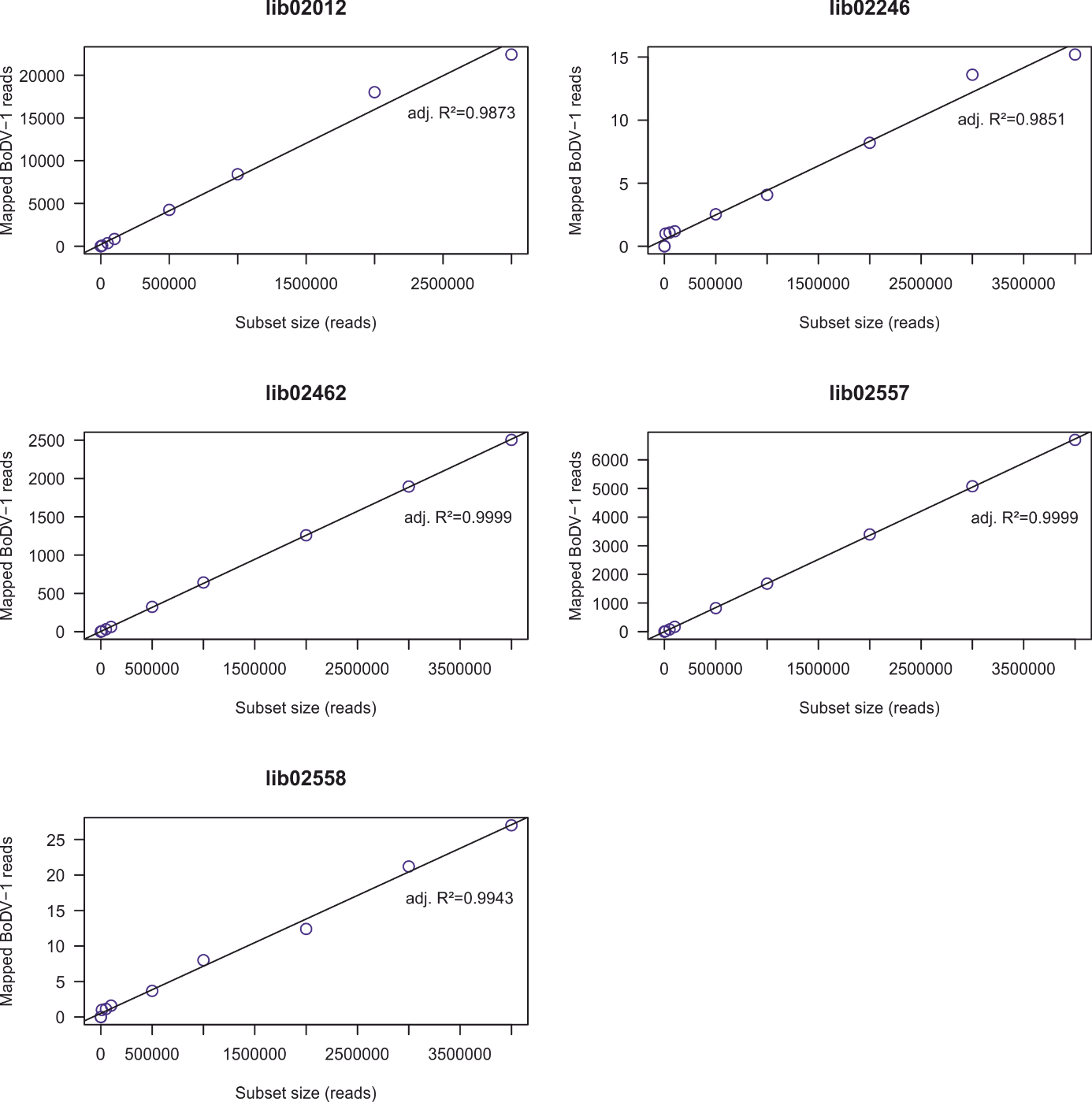


***Extended Data Figure 1.*** *Quantitative mapping results of BoDV-1 by rarefaction analysis. The mean of BoDV-1 reads per subsample were plotted versus subsample size.*

*
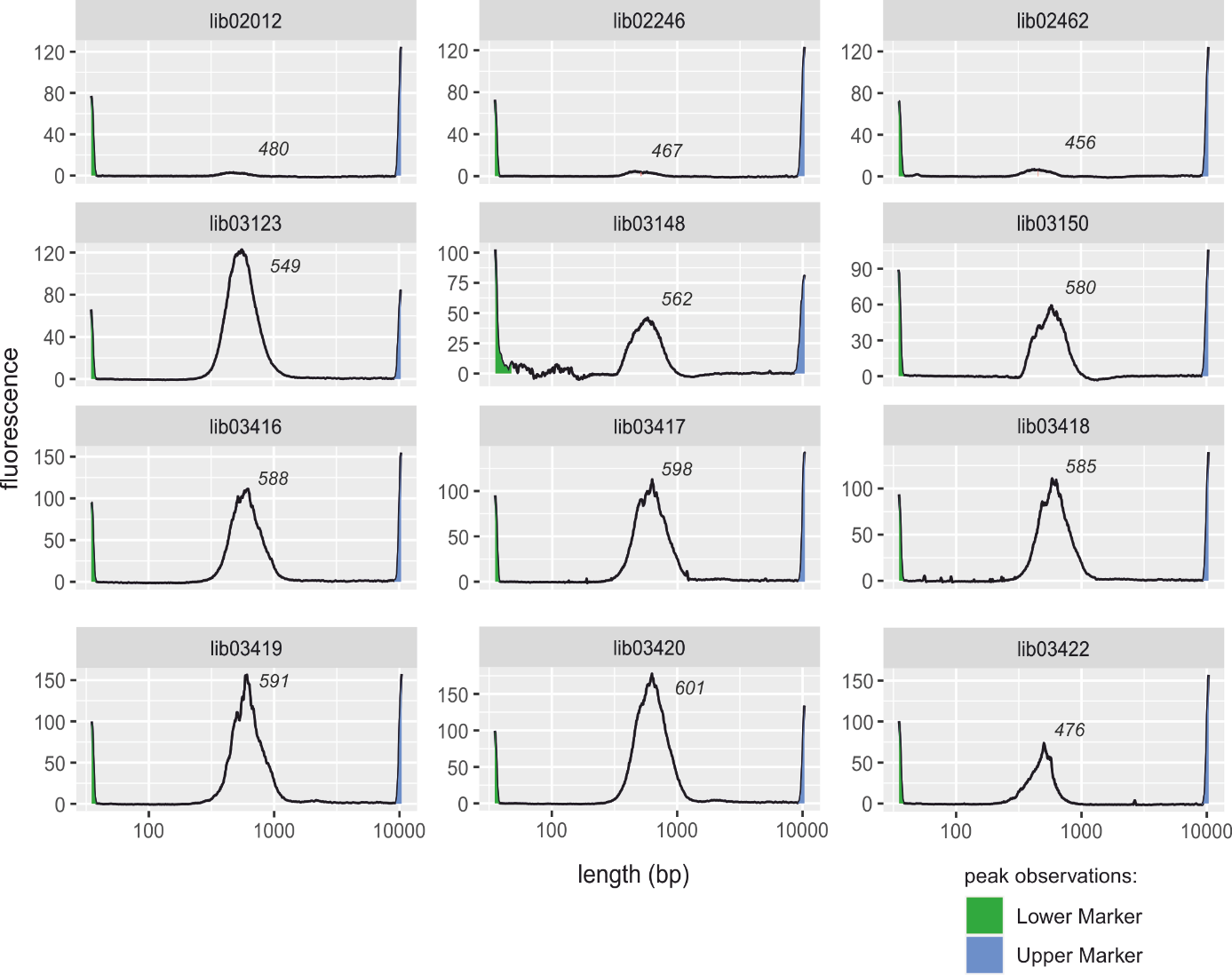
*

***Extended Data Figure 2.*** *Size distribution of exemplary libraries as determined by Bioanalyzer high-sensitivity DNA analysis. The numbers within the plots represent the mean size of the library.*


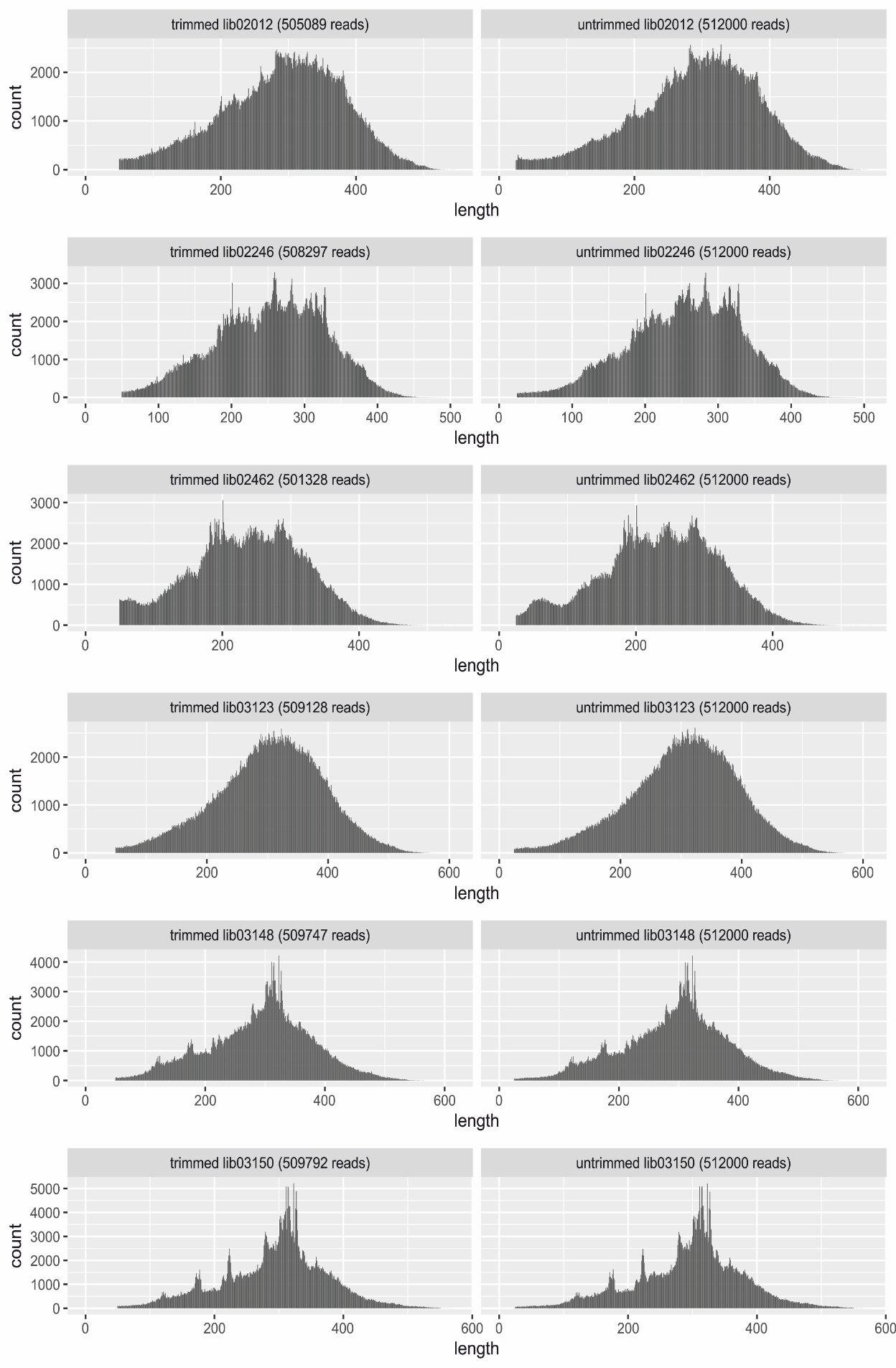

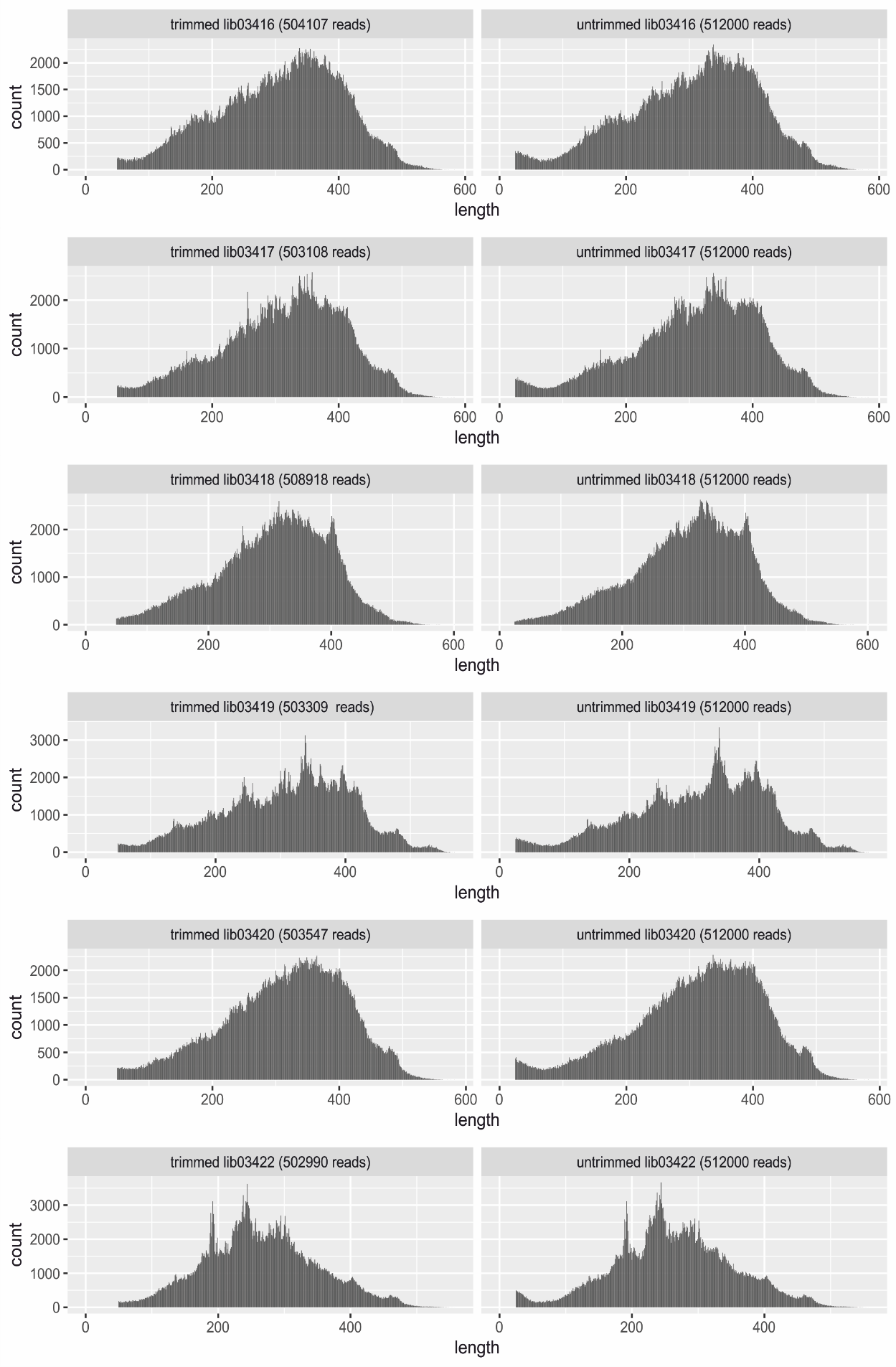


***Extended Data Figure 3.*** *Read length histogram of the sequencing results of exemplary libraries before and after quality and adapter trimming, a subset of 5.12E+05 reads were randomly extracted for each dataset prior to trimming.*


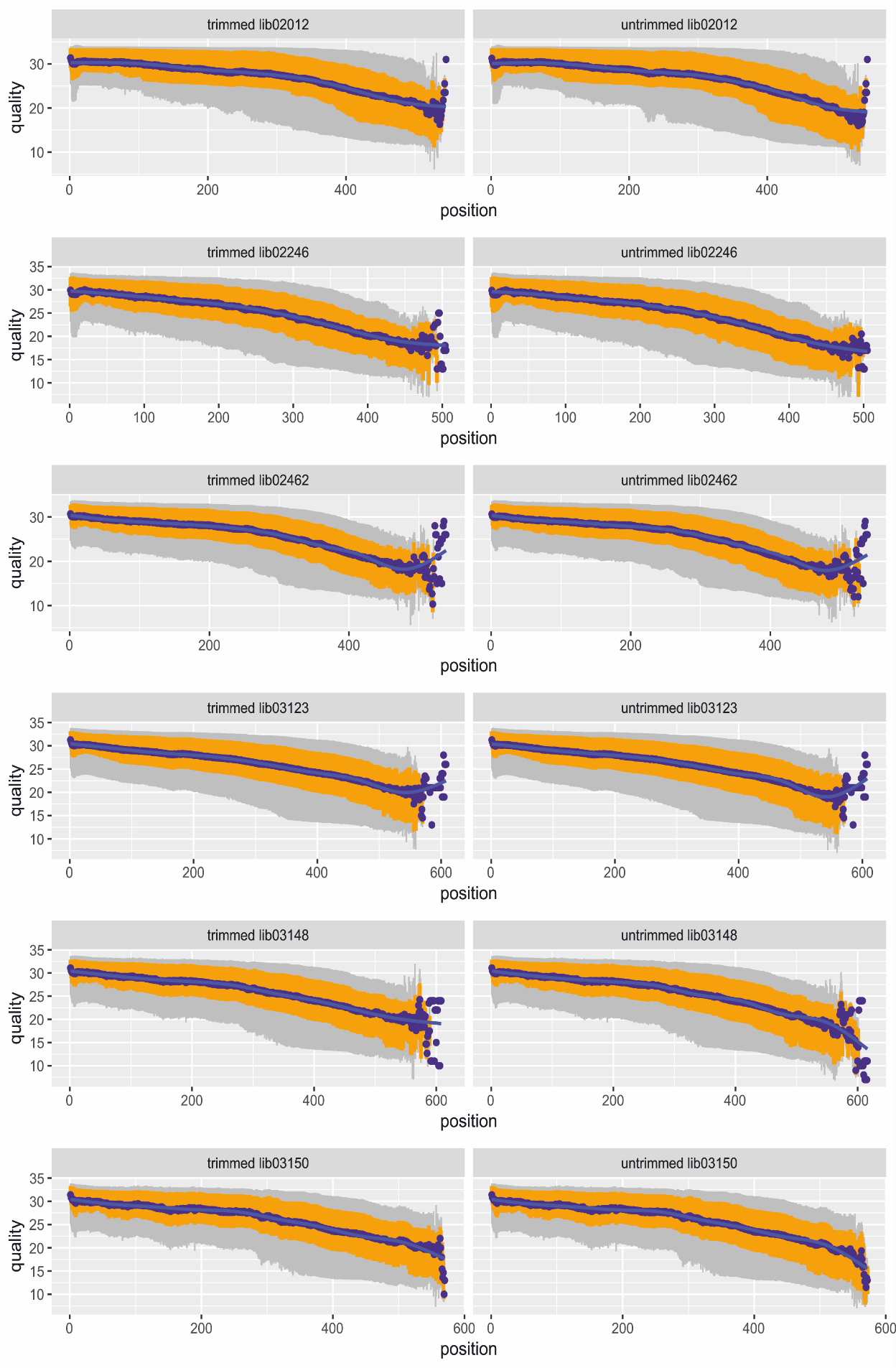

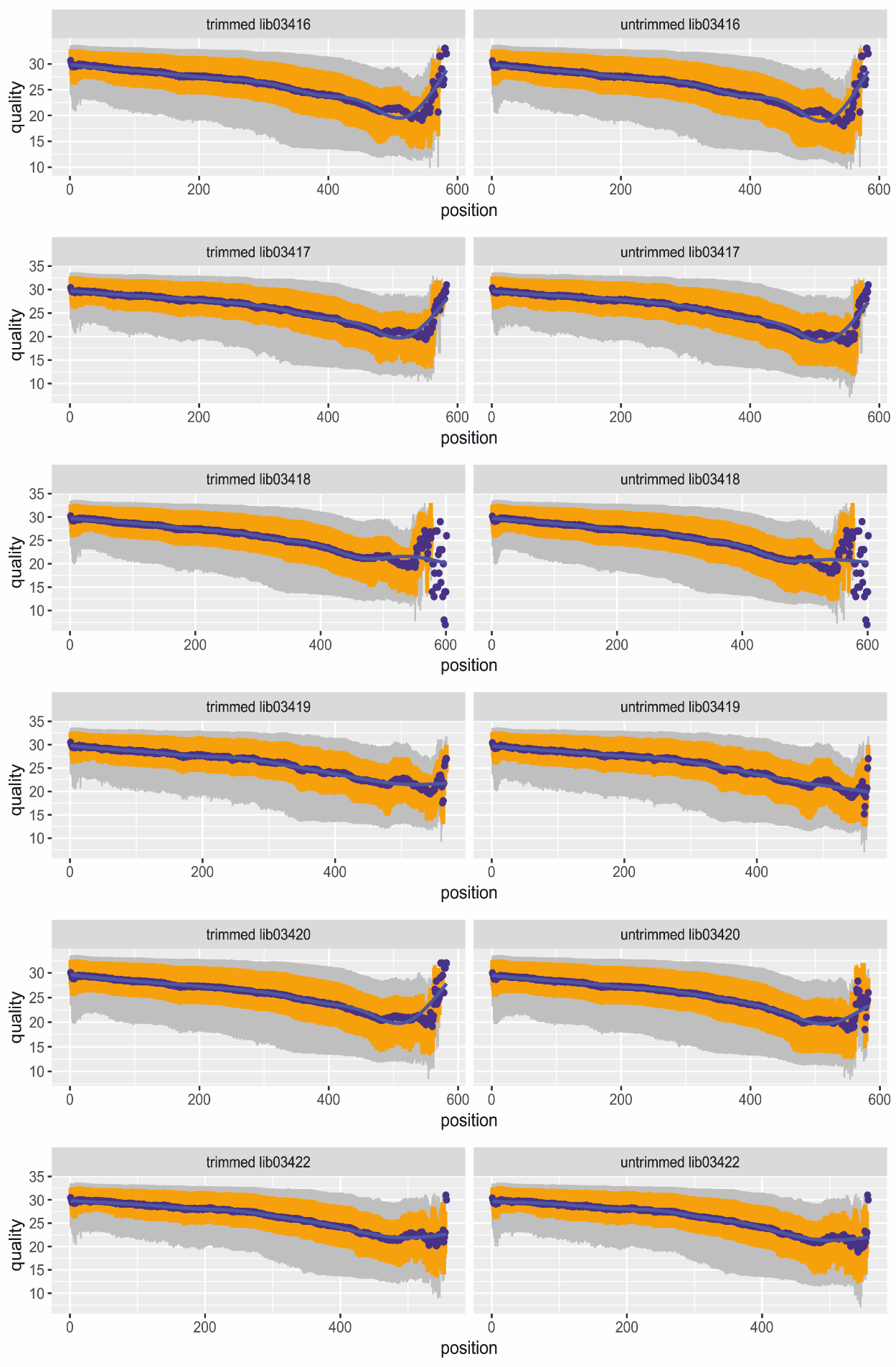


***Extended Data Figure 4.*** *Quality plots of the exemplary libraries before and after quality and adapter trimming.*


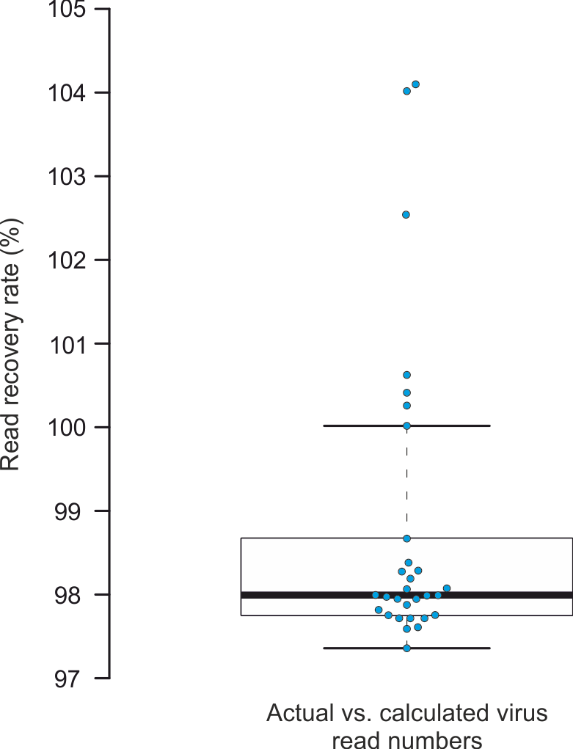


***Extended Data Figure 5.*** *Deviation of actual versus predicted viral read amounts as read recovery rate (%); median= 97.99%.*

***
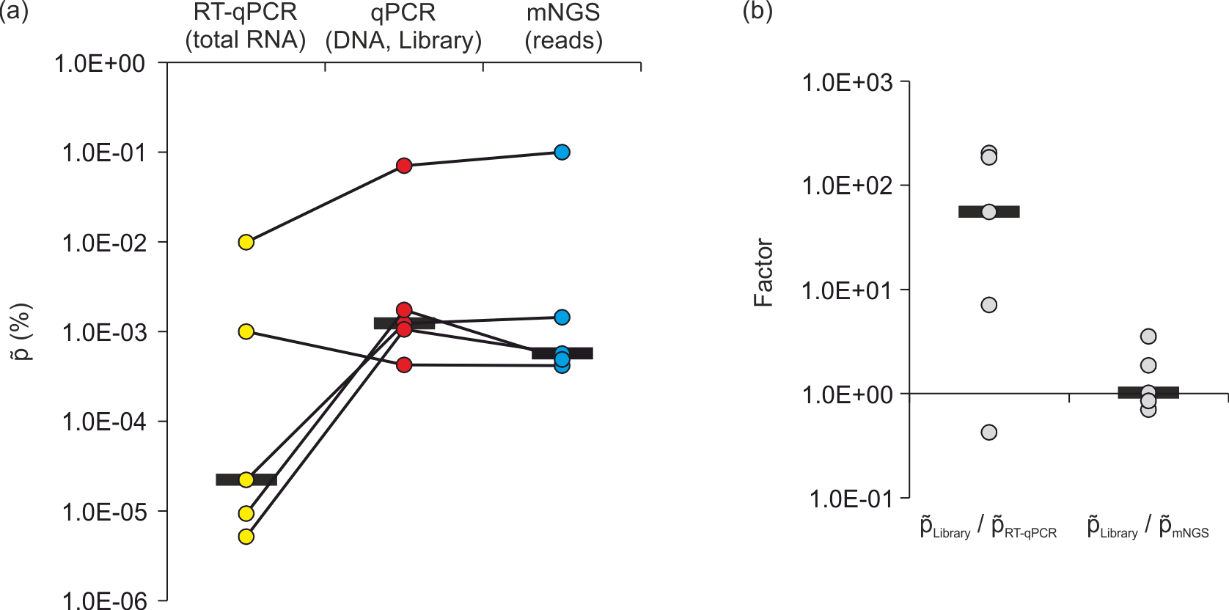
***

***Extended Data Figure 6.*** *Comparison of RT-qPCR, qPCR, and mNGS derived virus to background ratios (*p̃*). Five libraries were analyzed: lib02246, lib02462 (both BoDV-1), lib03123 (RusV), lib03148, and lib03150 (both PGV)*. (a) p̃ *were determined by absolute quantification by RT-qPCR (proportion of virus RNA in ng/µl to the total RNA concentration), by absolute quantification by qPCR (proportion of virus double-stranded cDNA in ng/µl to the library DNA concentration), and by mNGS analysis (proportion of virus reads to total reads).*

*(b) Discrepancy of qPCR, mNGS and RT-qPCR derived p̃ displayed as the factor of* p̃*_Library_ /* p̃*_RT-qPCR_ and* p̃*_Library_ /* p̃*_mNGS_ (median= 55.5, 1.0).*
